# Reduced mortality for over-the-scope clips (OTSC) versus surgery for refractory peptic ulcer bleeding: a retrospective study

**DOI:** 10.1007/s00464-022-09679-9

**Published:** 2022-10-17

**Authors:** Armin Kuellmer, Tobias Mangold, Dominik Bettinger, Moritz Schiemer, Julius Mueller, Andreas Wannhoff, Karel Caca, Edris Wedi, Tobias Kleemann, Robert Thimme, Arthur Schmidt

**Affiliations:** 1 Department of Medicine II, Medical Center, Faculty of Medicine, University of Freiburg, Hugstetter Strasse 55, 79106 Freiburg, Germany; 2 Department of Internal Medicine and Gastroenterology, Hospital Ludwigsburg, Ludwigsburg, Germany; 3 Medizinische Klinik II / IV, Sana Klinikum Offenbach, Offenbach, Germany; 4 Medinische Klinik IV, Carl-Thiem-Klinikum Cottbus, Cottbus, Germany

## Abstract

**Background:**

Surgery or transcatheter arterial embolization or are both considered as standard treatment of peptic ulcer bleeding (PUB) refractory to endoscopic hemostasis. Over-The-Scope clips (OTSC) have shown superiority to standard endoscopic treatment but a comparison with surgery has not been performed, yet.

**Patients and methods:**

In this retrospective, multicenter study, 103 patients treated with OTSC (*n* = 66) or surgery (*n* = 37) for refractory PUB in four tertiary care centers between 2009 and 2019 were analyzed. Primary endpoint was clinical success (successful hemostasis and no rebleeding within seven days). Secondary endpoints were adverse events, length of ICU-stay and in-hospital mortality. Univariable and multivariable regression models were performed to define predictive factors for allocation to surgical therapy and for mortality.

**Results:**

Age, comorbidities, anticoagulation therapy, number of pretreatments, ulcer location, and Rockall-Score were similar in both groups. In the surgical group, there were significantly more patients in shock at rebleeding (78.1% vs. 43.9%; *p* = 0.002), larger ulcers (18.6 ± 7.4 mm vs. 23.0 ± 9.4 mm; *p* = 0.017) and more FIa bleedings (64.9% vs. 19.7%; *p* < 0.001) were detected. Clinical success was comparable (74.2% vs. 83.8%; *p* = 0.329). In the surgical group, length of ICU-stay (16.2 ± 18.0 days vs. 4.7 ± 6.6 days; *p* < 0.001), severe adverse events (70.3% vs. 4.5%; *p* < 0.001) and in-hospital mortality (35.1% vs. 9.1%; *p* = 0.003) were significantly higher. Multivariable analysis defined shock at rebleeding as the main predictor for allocation to surgical therapy (OR 4.063, 95%CI {1.496–11.033}, *p* = 0.006). Postsurgical adverse events were the main reason for the in-hospital mortality (OR 5.167, 95% CI {1.311–20.363}, *p* = 0.019).

**Conclusion:**

In this retrospective study, OTSC compared to surgical treatment showed comparable clinical success but was associated with shorter ICU-stay, less severe adverse events and lower in-hospital mortality.

**Supplementary Information:**

The online version contains supplementary material available at 10.1007/s00464-022-09679-9.

Bleeding form peptic ulcers is still the most common cause of non-variceal upper gastrointestinal hemorrhage. While standard endoscopic therapy is effective in 90% of cases, the chance of achieving durable hemostastis drops with every additional rebleeding, ultimately leading to increased mortality [1]. In case of rebleeding after initial successful endoscopic therapy, a second endoscopic hemostasis attempt is recommended [2–4]. This recommendation is mainly attributable to the only RCT comparing endoscopic and surgical treatment in this indication [5]. In refractory cases, patients are regularly referred to other therapeutic modalities such as surgical treatment or transcatheter angiographic embolization (TAE). Meta-analyses and systematic reviews comparing these two modalities show a lower rebleeding rate for surgery yet a higher rate of complications. [6–8]. Although mortality was similar in these (older) meta-analyses, a recently published population-based study has found a lower long-term mortality in TAE patients [9]. With these conflicting results, the decision which therapy to choose relies on the treating physician, the local ressources and expertise. Over-the-scope clips (OTSC®, Ovesco endoscopy, Tuebingen, Germany) have shown high effectivity for severe PUB as demonstrated in multiple retrospective studies. One randomized study also demonstrated superiority over standard endoscopic treatment for recurrent bleeding [10–14]. Moreover, a recently published analysis comparing OTSC therapy against TAE in recurrent peptic ulcer bleeding found a reduced mortality and shorter ICU stay for OTSC [15] The purpose of this study was to compare OTSC therapy to surgery in refractory peptic ulcer bleeding of the upper gastrointestinal tract.

## Patients and methods

### Study design

Data of 1331 patients with bleeding from ulcers in the upper gastrointestinal tract at four hospital sites in Germany (University Hospital Freiburg, Ludwigsburg Hospital, University Medical Center Göttingen, Carl-Thiem Hospital Cottbus) were screened for eligibility. Inclusion criteria were: (a) bleeding from gastroduodenal peptic ulcers and (b) OTSC or surgery performed after failure of at least one endoscopic treatment (persistent or recurrent bleeding).

Exclusion criteria were: (a) bleeding of other source than peptic ulcer (e.g. Dieulafoy-lesion, variceal bleeding, anastomotic ulcers) and (b) execution of surgical or OTSC therapy without at least one prior endoscopic hemostasis attempt.

### Endpoints and definitions

The primary endpoint of the study was clinical success, defined as a combined endpoint of successful hemostasis and absence of rebleeding within seven days after the index intervention.

Secondary endpoints of our study were need for additional therapeutic intervention (“re-therapy”), length of hospital stay, length of stay on intensive care unit or intermediate care, need for red blood cell transfusion, adverse events and in-hospital mortality.

Rebleeding was defined using criteria as recommended [16] and/or if an intervention (endoscopic, radiographic, surgical) had to be performed for treatment.

Failure in the OTSC group was defined as inability to stop the bleeding after placement of the OTSC or if an OTSC could not be placed after the endoscope had already been loaded with the clip due to gastrointestinal stenosis or fibrotic tissue.

Adverse events and severe adverse events were defined according to good clinical practice guideline E6 (R2) (https://www.ich.org/page/efficacy-guidelines). Thus, a Serious Adverse Event (SAE) is any untoward medical occurrence that at any dose either a) results in death, b) is life-threatening c) requires inpatient ospitalization or prolongation of existing hospitalization and d) results in persistent or significant disability/incapacity.

Comorbidities were assessed using the Charlson comorbidity index [17].

### Search strategy

Specific diagnosis-codes for peptic ulcer bleeding and procedure-codes of OTSC or surgery of the German DRG-system were used for patient identification (see supplementary table 1). A list of patients fulfilling both criterions of diagnostic and procedural codes within a timeframe of 2009–2019 was created by searching in the respective hospital information system at each investigational site. The study was approved by the institutional review board of the University of Freiburg (No. 534/19; Dec 5th, 2019) and was performed in accordance with the Declaration of Helsinki.

### Data management and statistical analysis

Patients were recruited at their participating center and were integrated in local databases and summarized in a central database located at the University Medical Center Freiburg. The documentation of the patients from each center was within the responsibility of the local investigators. Baseline characteristics of the patients were analyzed at the time of recurrent peptic ulcer bleeding. Continuous variables are expressed as mean with standard deviation, whereas categorical variables are reported as frequencies and percentages unless stated otherwise. For continuous variables, differences were determined using Wilcoxon–Mann–Whitney and Kruskal–Wallis tests as there was no Gaussian distribution of the data confirmed by the Kolmogorov–Smirnov test. Χ^2^ tests or Fisher’s exact tests were used for categorical variables. *p* values < 0.05 were considered significant. Predictive factors for allocation to surgical treatment and analyses of predictive factors for in hospital mortality were analyzed using uni-and multivariable logistic regression models. Variables with a *p* < 0.05 in the multivariable model entered the multivariable model without further variable selection.

Data collection was performed with Microsoft © Excel 2016 for Mac Os (Version 15.21 (Microsoft, Redmond, USA). Statistical analyses were performed with SPSS (version 27.0, IBM, New York, USA).

## Results

### Baseline characteristics

**Fig. 1:**
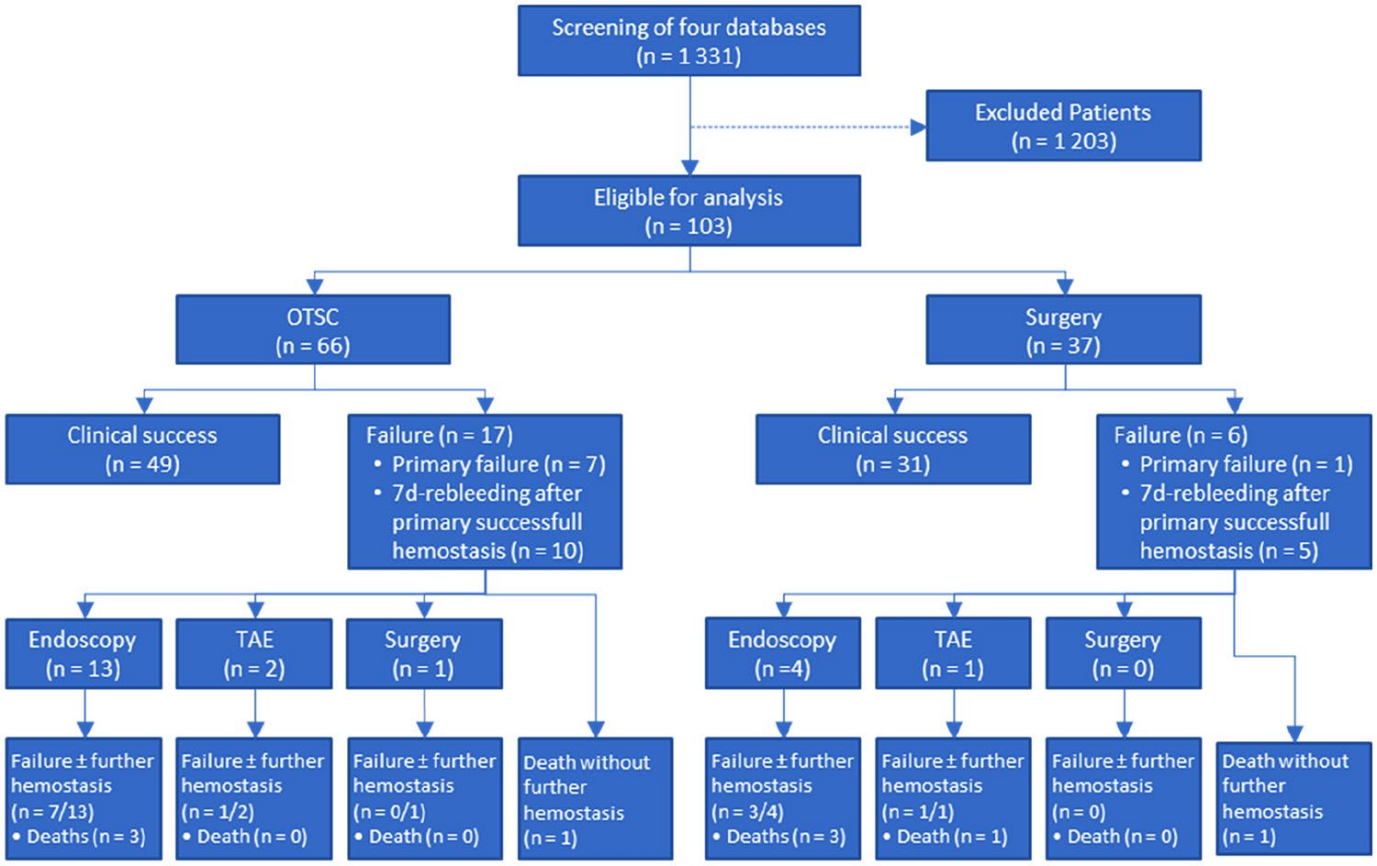
A flowchart of the study cohort is shown. Clinical success: successful hemostasis (no primary failure) AND the absence of a rebleeding within 7 days after intervention. Failure in the OTSC group: inability to stop the bleeding after placement of the OTSC, and/or if an OTSC could not be placed (after the endoscope was loaded with the clip). Failure of surgery: inability to stop a bleeding via surgical procedures

Medical records of 1331 patients were screened, 103 patients were eligible for further analysis (Fig. 1). OTSC therapy was performed in 66 patients and surgical treatment in 37 patients. Mean age in the OTSC group was 70.9 ± 13.4 years vs. 70.1 ± 11.3 years in the surgical group (*p* = 0.462). The mean Charlson comorbidity index (CCI) was 4.1 ± 2.7 and 3.7 ± 2.7 in the OTSC and surgical group, respectively (*p* = 0.491). In the OTSC group, 34/66 patients (51.5%) were on anticoagulation or anti-platelet therapy compared to 17/37 patients (45.9%) in the surgical group (*p* = 0.682). Table 1 summarizes the baseline characteristics of the included patients.

**Table 1:** Baseline characteristics of the total cohort are shown

	OTSC (*n* = 66)	Surgery (*n* = 37)	*p*
Patient characteristics			
Age (years), mean ± SD	70.9 ± 13.4	70.1 ± 11.3	0.462
Charlson Comorbidity Index, mean ± SD	4.0 ± 2.7	3.7 ± 2.7	0.491
Anticoagulation or platelet inhibition, *n* (%)	34 (51.5)	17 (45.9)	0.682
Bleeding characteristics			
Number of endoscopic pretreatments, mean ± SD	1.5 ± 0.7	1.7 ± 0.8	0.392
Hemoglobin before Salvage treatment {mg/}, mean ± SD	7.7 ± 1.6	6.6 ± 1.6	0.002
Shock at reebleding, *n* (%) (5 unknown in surgery)	29 (43.9)	25 (78.1)	0.002
Ulcer characteristics			
Size (mm), mean ± SD	18.6 ± 7.4	23.0 ± 9.4	0.017
Localisation			
Duodenal bulb *n* (%)	43 (65.2)	25 (67.6)	0.832
Other, *n* (%)	23 (34.8)	12 (32.4)	0.832
Forrest			
Forrest Ia, *n* (%)	13 (19.7)	24 (64.9)	< 0.001
Forrest Ib, *n* (%)	42 (63.6)	12 (32.4)	0.004
Forrest IIa, IIb, *n* (%)	11 (16.7)	1 (2.7)	0.052
Rockall score, mean ± SD	6.9 ± 1.8	6.9 ± 1.7	0.878

Statistical analysis was performed with Mann–Whitney *U*-test (continuous variables), and *χ*^2^ tests or Fisher’s Exact tests (categorial variables). *p* values < 0.05 were considered being significant

*OTSC* over-the-scope clips, *SD* standard deviation, *n* number

The mean number of endoscopic pretreatments was 1.5 ± 0.7 in the OTSC group and 1.7 ± 0.8 in the surgical group (*p* = 0.392). Mean hemoglobin level before therapy was higher in the OTSC group (7.7 mg/d*l* ± 1.6 versus 6.6 mg/d*l* ± 1.6; *p* = 0.002). Hemorrhagic shock at rebleeding was present in 29/66 patients (43.9%) in the OTSC group compared to 25/37 (78.1%) patients in the surgical group (*p* = 0.002).

Regarding ulcer characteristics, mean size (18.6 ± 7.4 mm vs. 23.0 ± 9.4 mm, *p* = 0.017) was significantly higher in the surgical group, yet the proportion of ulcers > 20 mm in size was not significantly different (27.3% vs. 45.9%; *p* = 0.082). The main localization of the bleeding ulcer was the duodenal bulb in both groups with no significant difference (65.2% vs. 67.6%; *p* = 0.832). Active bleedings (FIa and FIb) were present in 83.3% (OTSC) versus 97.3% (surgery) of cases (*p* = 0.052). Forrest Ia bleedings were observed in 13/66 of ulcers (19.7%) in the OTSC group in contrast to 24/37 of ulcers (64.9%) in the surgical group (*p* < 0.001), whereas FIb bleedings were significantly more frequent in the OTSC group (42/66 of patients (63.6%) versus 12/37 of patients (32.4%; *p* = 0.004). The mean Rockall score in both groups was 6.91 ± 1.8 in the OTSC group and 6.9 ± 1.7 in the surgical group (*p* = 0.878).

Oversewing was the main surgical treatment, accounting for 49% of patients (18/37) followed by Billroth-I-resection in 22% (8/37), (sub) total gastrectomy with roux-en-y reconstruction in 22% (8/37), wedge-resection in 5% (2/37) and Billroth-II-resection in 3% (1/37).

### Outcome

Clinical success was achieved in 74.2% of the OTSC group as opposed to 83.8% in the surgical group (*p* = 0.329). Primary success (89.4% vs. 97.3%; *p* = 0.253) and 7-day rebleeding rate (15.2% vs. 13.5%; *p* = 0.999) were also similar in both groups. The number of re-therapies were similar in both groups. However, in the OTSC group, re-therapy could be performed endoscopically in the majority of cases (71.1%; 27/38 procedures). TAE was done in 15.8% (6/38 procedures) and surgery in 13.2% (5/38 procedures) of cases. In contrast, surgery was the main modality in the surgical group, accounting for 76.7% (33/43) of procedures. Endoscopic re-therapy was performed in 14,0% (6/43) and re-TAE in 9.3% (4/43) of cases. In comparison, surgical re-therapy was performed significantly more frequently in the surgical group (*n* = 33 vs. *n* = 5; *p* < 0.001). Outcome parameters are summarized in Table 2.

**Table 2:** Outcome parameters are shown

	OTSC	*n* = 66	Surgery	*n* = 37	*p*	OR	95%-Confidence-Interval	
Clinical success, *n*, (%)	49	(74.2)	31	(83.8)	0.329	1.79	[0.64–5.04]	
Primary success, *n*, (%)	59	(89.4)	36	(97.3)	0.253	4.27	[0.50–36.15]	
7-day rebleeding, *n*, (%)	10	(15.2)	5	(13.5)	0.999	0.88	[0.27–2.79]	
Need for re-therapy, *n*, (%)	20	(30.3)	17	(45.9)	0.136	1.96	[0.85–4.50]	
Number of re-therapy, *n*, /patient	38		43		0.073			
Re-Endo, *n*, % of re-therapy	27	(0.71)	6	(0.14)	0.148			
Re-TAE, *n*, % of re-therapy	6	(0.16)	4	(0.09)	0.719			
Surgery, *n*, % of re-therapy	5	(0.13)	33	(0.77)	< 0.001			
Severe adverse events, OTSC/surgery related; *n*, (%)	2	(3.0)	26	(70.3)	< 0.001			
Severe adverse events, re-therapy-related; *n*, (%)	1	(1.5)	0		0.999			
Severe adverse events, OTSC/surgery and re-therapy-related; *n*, (%)	3	(4.5)	26	(70.3)	< 0.001			
Red blood cell transfusions, mean ± SD	3.7	(6.5)	5.4	(8.9)	0.906			
Length of hospital stay {days}, mean ± SD	15.2	± 12.4	23.1	± 18.4	0.001			
Lenght of ICU or IMC {days}, mean ± SD	4.7	± 6.6	16.2	± 18.0	< 0.001			
In-hospital mortality, *n*, (%)	6	(9.1)	13	(35.1)	0.003	5.42	[1.85–15.90]	

Statistical analysis was performed with Mann–Whitney U-Test (continuous variables), and χ^2^ tests or Fisher’s Exact tests (categorial variables). *p* values < 0.05 were considered being significant

*OTSC* over-the-scope clips, *SD* standard deviation, *n* number, *OR* Odds ratio, *CI* confidence interval

Severe adverse events (SAE) occurred in 4.5% of patients the OTSC group (*n* = 3) and in 70.3% of the surgical group (*n* = 26) (*p* < 0.001). These consisted of tissue irritation of the clip leading to hemorrhage in two cases and one insufficiency of a duodenal stump after surgical re-therapy in the OTSC group. In the surgical group, all of the SAEs (*n* = 26) were surgery-related. Main SAEs in the surgical group were suture or stapler insufficiencies, accounting for 50% of SAEs. In detail, these were mainly anastomotic insufficiencies (27%) followed by insufficiencies of gastrotomies/duodenotomies (12%) or a duodenal stump (12%). Other SAEs in this group were wound dehiscence (27%), paralytic ileus (15%) and tissue ischemia (8%). Mean number of red blood cell transfusions was comparable in both groups (3.7 ± 6,5 vs. 5.4 ± 8.9; *p* = 0.906). The length of hospital stay was significantly longer in the surgical group (23.1 ± 18.4 days vs. 15.2 ± 12.4 days; *p* = 0.001) as well as duration of ICU treatment (16.2 ± 18 days vs. 4.7 ± 6.6 days; *p* < 0.001).

The in-hospital mortality in the OTSC group was significantly lower than in the surgical group (9.1% versus 35.1%; *p* = 0.003; OR 5.42 [95% CI 1.85–15.90]).

### Factors associated with allocation to surgical treatment

In order to determine predictive factors for allocation to surgical treatment, uni- and multivariable logistic regression models were performed (Table 3). Multivariable logistic regression model identified shock at rebleeding (OR 4,263, 95% CI {1.545–11.746}; *p* = 0.005) and ulcer size (OR 1.090, 1.020–1.165; *p* = 0.011) as independent predictors for allocation to surgical treatment. Comorbidities as well as primary success during the first bleeding episode did not emerge as significant and independent predictive factors.

**Table 3:** Univariable and multivariable logistic regression regarding allocation to surgical treatment are shown

Parameter	Univariable model				Multivariable model			
	*β*	OR	95%CI	*p* value	*β*	OR	95%CI	*p* value
Primary success in firstline	− 0.925	0.397	0.173–0.908	0.029	0.917	2.502	0.276–22.660	0.415
Shock at rebleeding	1.517	4.557	1.729–12.007	0.002	1.449	4.260	1.545–11.746	0.005
Ulcer size	0.067	1.069	1.012–1.129	0.017	0.087	1.090	1.020–1.165	0.011
Charlson comorbidity Index (full score)	− 0.051	0.95	0.817–1.105	0.508				

*β* regression coefficient, *OR* Odds ratio, *95% CI* 95% confidence interval

### Subgroup analysis of patients with shock at re-bleeding

Hemorrhagic shock was the most important factor for allocation to surgical treatment. Patients with shock at the re-bleeding event and who received OTSC application had similar clinical success rates (75.9% vs. 80.0%, *p* = 0.755) and rebleeding rates during the hospital stay (17.2% vs. 20.0%, *p* = 0.999), 7 days (17.2% vs. 16.0%, *p* = 0.999) and 30 days (17,2% vs. 20.0%, *p* = 0.999) after the re-bleeding episode. Importantly, in-hospital mortality was significantly higher in patients with shock allocated to surgical treatment (6.9% vs. 32.0%, *p* = 0.032). Data is also shown in the supplemental material Table 2.

### Factors associated with increased in-hospital mortality

A total number of *n* = 19 patients (18%) died within the patient cohort.

During the hospital stay six patients (9.1%) died in the OTSC group compared to 13 (35.1%) patients in the surgery group (*p* =). While seven-day mortality was not significantly different in both groups (3% vs. 2.7%; *p* = 0.99), 30-day mortality was significantly higher in the surgery group compared to the OTSC group (7.6% vs. 24.3%, *p* = 0.033). Multivariable logistic regression model for analysis of predictive factors for in-hospital mortality identified adverse events after therapy (OR 5.167, [95% CI 1.311–20.363]; *p* = 0.019), allocation to surgical treatment (OR 4,161, 95% CI {1,129–15,346}, *p* = 0.032) and CCI (as independent significant risk factors for index mortality. Table 4 shows the uni- and multi-variable analysis.

**Table 4:** Univariable and multivariable logistic regression regarding mortality to surgical treatment are shown

Parameter	Univariable model				Multivariable model			
	*β*	HR	95%CI	*p* value	*β*	HR	95%CI	*p* value
Group	1.689	5.417	1.845–15.902	0.002	1.426	4.161	1.129–15.346	0.032
Shock at rebleeding	0.573	1.773	0.557–5.637	0.332				
Charlson Comorbidity Index (full score)	0.235	1.264	1.051–1.52	0.013	0.31	1.36	1.087–1.698	0.007
Coagulation disorder	2.279	9.765	0.837–113.896	0.069				
Primary success in firstline	− 0.38	0.684	0.251–1.863	0.457				
Coagulation abnormalities at rebleeding	− 0.048	0.953	0.305–2.985	0.935				
Ulcus cat size	− 0.442	0.643	0.211–1.959	0.437				
Forrest Ia	0.588	1.8	0.657–4.934	0.253				
Forrest Ib	0.01	1.01	0.373–2.738	0.984				
Forrest IIa	− 19.871	0	0–0	0.999				
Complication second line	2.15	8.55	2.685–27.228	< 0.001	1.642	5.167	1.311–20.363	0.019

*β* regression coefficient, *HR* Harzard ratio, *95% CI* 95% confidence interval

## Discussion

Refractory peptic ulcer bleeding is still a common problem in interventional endoscopy with significant morbidity and mortality. Our retrospective study demonstrates that treatment with over-the-scope clips (OTSC) has comparable technical and clinical success compared to surgery. It is associated with shortened stay on ICU and lower complication rates, leading to a lower in-hospital mortality. To our knowledge, this is the first study directly comparing OTSC and surgery for refractory PUB.

To date, there is only one RCT comparing standard endoscopic therapy to surgery in terms of peptic ulcer bleeding [5]. Success rates of endoscopic therapy are similar, yet the study is not fully comparable to our data for several reasons: different hemostasis methods were used (thermal and injection vs. OTSC ± injection) and the mean time to rebleeding was shorter (72 h vs. 7 day in our study)., Moreover, only rebleeding patients after a single endoscopic hemostasis attempt were included (our study also included persistent bleedings and patients with numerous previous hemostasis attempts). Furthermore, number of transfusions or length of hospital stay are not comparable to current studies as patient management including transfusion strategies changed markedly over the last 22 years. Our study results are in concordance with the finding that severe adverse events were significantly higher in the surgical group. Mortality was almost doubled in the surgical group (18% vs. 10%) yet did not reach statistical significance, most likely due to low patient numbers.

With respect to baseline characteristics in our analysis, the cohort seems comparable to meta-analyses on OTSC [11, 13] and surgical studies [6, 8, 18]. However, the mean Charlson Comorbidity Index in our study is higher compared to other studies [7, 9, 18, 19].

There were no significant differences in both groups regarding age, CCI, patients on anticoagulation or platelet inhibition, number of pretreatments, ulcer location (65% duodenal bulb), ulcer size above 20 mm and Rockall Score. However, the proportion of F1a bleedings, mean ulcer size and patients with hemodynamic instability was significantly higher in the surgery group. As all three factors have been shown to be predictors of endoscopic treatment failure we cannot exclude that may have biased the results in favour of OTSC treatment.[2, 5, 20]. However, available studies indicate that endoscopic treatment of severe hemorrhage (including FIa bleeding) with OTSC is highly effective [11, 21–23] The compression force of the clip is significantly higher compared to standard clips and burst pressures are similar to a surgical suture as animal studies have shown [24, 25]. In our clinical experience, as soon as the bleeding vessel can be detected and reached with the endoscope, achieving hemostasis with an OTSC is possible in almost all cases irrespective of a spurting or oozing lesion. This is also supported by the fact that the success rate of OTSCs is higher when applied to a persistent bleeding rather than a recurrent bleeding [11, 23]. Second, factors associated with failure of OTSC treatment is scarce, which makes a comparison to risk factors associated with failure of standard treatment difficult. While Elmunzer et al. found active bleeding being a risk factor for rebleeding in standard therapy [2], Richter-Schrag [12]did not find a significant link between active and non-active bleedings regarding the outcome of OTSC therapy (only FLET vs. SLET was significant). In the STING study, neither Forrest Ia nor ulcer size > 20 mm were risk factors for treatment failure on univariate analysis [11]. Third, in a study investigating OTSC in patients with anticoagulation, the proportion of rebleeders with FIa and Fib bleedings was not different [26]. On the other hand, Wedi et al. found a significant association between Forrest classification and risk of rebleeding but only investigated patients with first-line OTSC [27].

Regarding patients in shock at rebleeding, multivariate analysis of our patient cohort showed that along with ulcer size this was the main allocation criteria to surgical therapy (Table 3). The reason for this could not be cleared, as the number of pretreatments and ulcer location were not different in both groups. Larger ulcers (mean) may have led to more intense bleedings, yet the proportion of ulcers > 20 mm in size in both groups was not significantly different in both groups.

Clinical success, the primary endpoint of the study, was comparable in both groups with 74.2% in the OTSC group vs. 83,8% in the surgical group (*p* = 0.329). Regarding primary success and re-bleeding in the OTSC group, results are in line with most other studies investigating OTSC therapy for severe upper GI-bleeding with the restriction that usually first-line therapy and various bleeding entities were analyzed [13, 14]. In contrast to its high efficacy as first line therapy, success rates of OTSC therapy drop when used as a second-line or salvage therapy [11–14, 27]. Moreover, second-line OTSC therapy compared to first-line OTSC is an independent risk factor for rebleeding in multivariable analysis [12].

It is known that ulcer-related and clinical parameters such as comorbidities increase the risk of rebleeding, irrespective of the treatment modalities [2, 28, 29]. With a higher mean Charlson comorbidity index in comparison to other studies, the failure rate in our study of 26% seems appropriate.

Regarding the surgical group, achieving hemostasis is regularly nearby 100%, so the rebleeding rate accounts for clinical success. The rebleeding rate of 13.5% in our study matches with three meta-analysis reporting around 15% [6–8].

The in-hospital mortality rate in our study was significantly higher in the surgical compared to the OTSC group (35.1% vs. 9.1%; OR 5.42 [95% CI: 1.85–15.90]; *p* = 0.003) (See also Table 2). Only two studies using OTSC as second-line or salvage therapy have reported mortality rates. It ranges from 9.1% in the STING study up to 27% in a retrospective study by Richter-Schrag et al., but it has to be noted that the latter is hardly comparable to our study as it included various indications of OTSC therapy (e.g. bleeding anastomotic ulcers and malignancies) [11, 12]. Moreover, chronic comorbidities were not addressed systematically in both studies. Mortality rates in the surgical studies vary between 10 and 50% of cases [6–8], but if only studies with a reasonable number of deaths recorded (*n* ≥ 10) are counted, the number increases from 20 up to 50% of cases [28, 30, 31]. Our reported 35**%** is in the upper half of this scale, but it has to be noted that our study cohort had a higher mean CCI, which is a validated prognostic factor regarding mortality.

To further clarify why surgical patients display a significantly higher mortality, univariate and multivariate regression analysis was performed: independent risk factors were CCI, surgical therapy and adverse events after re-therapy. In other words, the strongest predictor for death in this analysis were adverse events arising from surgical therapy leading to further interventions. The rate of 70.3% of SAEs match with the reported rate of 60% in the analysis of Nykänen et al. Mainly anastomotic or stapler insufficiencies, not rebleeding, were the reason for re-therapy in the surgical cohort. This also explains why re-therapy in the surgical group had to be surgical in 77% of cases while surgery in the OTSC group was necessary in 13% (*p* < 0.001).

Shock and severe anemia/need for transfusion are known risk factors for anastomotic insufficiency after surgery [32–34]. The high percentage of patients in shock at rebleeding in the surgical group together with the high rate of insufficiencies leading to complications supports this finding. Taken together, surgical (re-) treatment should be avoided in these usually frail and multimorbid patients as mortality rises due to a higher risk of anastomotic insufficiency, which is in part a consequence of shock resulting from uncontrolled bleeding. From our point of view, OTSC treatment should therefore be attempted in every suitable case to prevent this deadly cascade before a patient is sent to surgery.

Our study has several limitations: the main weakness is the retrospective design of the study. Patients were not randomly assigned to OTSC or surgical therapy but decision on therapy was rather based on decision of the endoscopist or surgeon which makes an inclusion bias possible. A second limitations is that treatment itself did not follow a standard protocol. A third limitation is the difference in baseline characteristics regarding shock and proportions of FIa bleedings. Although doubts can be expressed regarding the generalizability of rebleeding risk factors regarding OTSC treatment, shock is unquestionable an indicator of poor outcome and the difference in both groups limit the comparability of the two groups.

## Conclusion

In this study, OTSC compared to surgical treatment in refractory PUB showed comparable efficacy in terms of hemostasis but was associated with a lower in hospital mortality and shorter ICU stay. Multivariable analysis defined charlson comorbidity index and postsurgical complications as main predictors for in-hospital mortality. Our analysis indicates that endoscopic treatment with OTSC should be preferred over surgery for refractory PUB.

The retrospective design of the study and differences in baseline characteristics (e.g. higher number of patients in shock in surgical group) may limit the generizability of the results. A randomized controlled trial could define risk factors associated with failure of OTSC treatment in this indication and to reduce the risk of selection bias. However, due to ethical reasons and in terms of feasibility, this would be almost impossible to perform.

## Supplementary Information

Below is the link to the electronic supplementary material.

Supplementary file1 (DOCX 20 kb)

## References

[1] Laine L, Jensen DM (2012). Management of patients with ulcer bleeding. Am J Gastroenterol.

[2] Elmunzer BJ, Young SD, Inadomi JM, Schoenfeld P, Epi MS, Laine L (2008). Systematic review of the predictors of recurrent hemorrhage after endoscopic hemostatic therapy for bleeding peptic ulcers. Am J Gastroenterol.

[3] Götz M, Anders M, Biecker E, Bojarski C, Braun G, Brechmann T, Dechêne A, Dollinger M, Gawaz M, Kiesslich R, Schilling D, Tacke F, Andus T, Appenrodt B, Aschoff A, Benten D, Caca K, Denzer U, Diepolder H, Fischbach W, Gebauer B, Gerbes AL, Gülberg V, Hohn H, Jakobs R, Juchems M, Jung M, Keuchel M, Klamroth R, Leyhe T, Lynen-jansen P, Meining A, Messmann H, Metzger R, Mudter J, Neuhaus H, Rey JW, Riphaus A, Roeb E, Salomon F, Schaible A, Schultheiß M, Sibbing D, Simon A, Strassburg CP, Pfeh EM, Ng LU (2017) S2k-Leitlinie Gastrointestinale Blutung S2k Guideline Gastrointestinal Bleeding Guideline of the German Society of Gastroenterology DGVS Authors Einleitung Arbeitsgruppe 1: Prä-endoskopisches. 883–936

[4] Gralnek IM, Stanley AJ, Morris AJ, Camus M, Lau J, Lanas A, Laursen SB, Radaelli F, Papanikolaou IS, Cúrdia Gonçalves T, Dinis-Ribeiro M, Awadie H, Braun G, De Groot N, Udd M, Sanchez-Yague A, Neeman Z, Van Hooft JE (2021). Endoscopic diagnosis and management of nonvariceal upper gastrointestinal hemorrhage (NVUGIH): European Society of Gastrointestinal Endoscopy (ESGE) Guideline—Update 2021. Endoscopy.

[5] Lau JY, Sung JJ, Lam YH, Chan AC, Ng EK, Lee DW, Chan FK, Suen RC, Chung SS (1999). Endoscopic retreatment compared with surgery in patients with recurrent bleeding after initial endoscopic control of bleeding ulcers. N Engl J Med.

[6] Beggs AD, Dilworth MP, Powell SL, Atherton H, Griffiths EA (2014). A systematic review of transarterial embolization versus emergency surgery in treatment of major nonvariceal upper gastrointestinal bleeding. Clin Exp Gastroenterol.

[7] Darmon I, Rebibo L, Diouf M, Chivot C, Riault C, Yzet T, Le Mouel JP, Regimbeau JM (2020). Management of bleeding peptic duodenal ulcer refractory to endoscopic treatment: surgery or transcatheter arterial embolization as first-line therapy? A retrospective single-center study and systematic review. Eur J Trauma Emerg Surg.

[8] Kyaw M, Tse Y, Ang D, Ang T, Lau J (2014). Embolization versus surgery for peptic ulcer bleeding after failed endoscopic hemostasis: a meta-analysis. Endosc Int Open.

[9] Sverdén E, Mattsson F, Lindström D, Sondén A, Lu Y, Lagergren J (2019). Transcatheter arterial embolization compared with surgery for uncontrolled peptic ulcer bleeding: a population-based cohort study. Ann Surg.

[10] Prosst RL, Kratt T (2017). A randomized comparative trial of OTSC and Padlock for upper GI hemostasis in a standardized experimental setting. Minim Invasive Ther Allied Technol.

[11] Schmidt A, Gölder S, Goetz M, Meining A, Lau J, von Delius S, Escher M, Hoffmann A, Wiest R, Messmann H, Kratt T, Walter B, Bettinger D, Caca K (2018). Over-the-scope clips are more effective than standard endoscopic therapy for patients with recurrent bleeding of peptic ulcers. Gastroenterology.

[12] Richter-Schrag HJ, Glatz T, Walker C, Fischer A, Thimme R (2016). First-line endoscopic treatment with over-the-scope clips significantly improves the primary failure and rebleeding rates in high-risk gastrointestinal bleeding: a single-center experience with 100 cases. World J Gastroenterol.

[13] Chandrasekar VT, Desai M, Aziz M, Patel HK, Gorrepati VS, Jegadeesan R, Rai T, Sathyamurthy A, Murino A, Hassan C, Repici A, Sharma P (2019). Efficacy and safety of over-the-scope clips for gastrointestinal bleeding: a systematic review and meta-analysis. Endoscopy.

[14] Ofosu A, Ramai D, John F, Barakat M, Sunkara T, Sharma S, Gaduputi V, Adler DG, Reddy M (2019). Over-the-scope-clips as primary and rescue therapy for non-variceal gastrointestinal bleeding: a systematic review and meta-analysis. Minerva Gastroenterol Dietol.

[15] Kuellmer A, Mangold T, Bettinger D, Maruschke L, Wannhoff A, Caca K, Wedi E, Hosseini ASA, Kleemann T, Schulz T, Jung C, Thimme R, Schmidt A (2021). Over-the-scope clip versus transcatheter arterial embolization for refractory peptic ulcer bleeding—a propensity score matched analysis. United Eur Gastroenterol J.

[16] Laine L, Spiegel B, Rostom A, Moayyedi P, Kuipers EJ, Bardou M, Sung J, Barkun AN (2010). Methodology for randomized trials of patients with nonvariceal upper gastrointestinal bleeding: recommendations from an International Consensus Conference. Off J Am Coll Gastroenterol.

[17] Charlson ME, Pompei P, Ales KL, MacKenzie CR (1987). A new method of classifying prognostic comorbidity in longitudinal studies: development and validation. J Chronic Dis.

[18] Nykänen T, Peltola E, Kylänpää L, Udd M (2017). Bleeding gastric and duodenal ulcers: case-control study comparing angioembolization and surgery. Scand J Gastroenterol.

[19] Laursen SB, Jakobsen M, Nielsen MM, Hovendal C, Schaffalitzky De Muckadell OB (2014). Transcatheter arterial embolization is the first-line therapy of choice in peptic ulcer bleeding not responding to endoscopic therapy. Scand J Gastroenterol.

[20] Rockall TA, Logan RFA, Devlin HB, Northfield TC (1996). Risk assessment after acute upper gastrointestinal haemorrhage. Gut.

[21] Manta R, Mangiafico S, Zullo A, Bertani H, Caruso A, Grande G, Zito FP, Mangiavillano B, Pasquale L, Parodi A, Germanà B (2018). First-line endoscopic treatment with over-the-scope clips in patients with either upper or lower gastrointestinal bleeding: a multicenter study. Endosc Int Open.

[22] Jensen DM, Kovacs T, Ghassemi KA, Kaneshiro M, Gornbein J (2021). Randomized controlled trial of over-the-scope clip as initial treatment of severe nonvariceal upper gastrointestinal bleeding. Clin Gastroenterol Hepatol.

[23] Meier B, Wannhoff A, Denzer U, Stathopoulos P, Schumacher B, Albers D, Hoffmeister A, Feisthammel J, Walter B, Meining A, Wedi E, Zachäus M, Pickartz T, Küllmer A, Schmidt A, Caca K (2022) Over-the-scope-clips versus standard treatment in high-risk patients with acute non-variceal upper gastrointestinal bleeding: a randomised controlled trial (STING-2). Gut gutjnl-2021-325300. 10.1136/gutjnl-2021-325300 10.1136/gutjnl-2021-325300 35321938

[24] von Renteln D, Schmidt A, Vassiliou MC, Gieselmann M, Caca K (2009). Natural orifice transluminal endoscopic surgery gastrotomy closure with an over-the-endoscope clip: a randomized, controlled porcine study (with videos). Gastrointest Endosc.

[25] von Renteln D, Rudolph HU, Schmidt A, Vassiliou MC, Caca K (2010). Endoscopic closure of duodenal perforations by using an over-the-scope clip: a randomized, controlled porcine study. Gastrointest Endosc.

[26] Lamberts R, Koch A, Binner C, Zachäus M, Knigge I, Bernhardt M, Halm U (2017). Use of over-the-scope clips (OTSC) for hemostasis in gastrointestinal bleeding in patients under antithrombotic therapy. Endosc Int Open..

[27] Wedi E, Fischer A, Hochberger J, Jung C, Orkut S, Richter-Schrag HJ (2018). Multicenter evaluation of first-line endoscopic treatment with the OTSC in acute non-variceal upper gastrointestinal bleeding and comparison with the Rockall cohort: the FLETRock study. Surg Endosc.

[28] Wong TCL, Wong KT, Chiu PWY, Teoh AYB, Yu SCH, Au KWL, Lau JYW (2011). A comparison of angiographic embolization with surgery after failed endoscopic hemostasis to bleeding peptic ulcers. Gastrointest Endosc.

[29] Sung JJYY, Chiu PCYWYCY, Chan FKLL, Lau JYWW, Goh KL, Ho LHYY, Jung HY, Sollano JD, Gotoda T, Reddy N, Singh R, Sugano K, Wu KC, Wu CY, Bjorkman DJ, Jensen DM, Kuipers EJ, Chen M, Ching JYL, Ho KY, Kachintorn U, Kim N, Lau JYWW, Menon J, Rani AA, Reddy N, Sollano JD, Sugano K, Tsoi KKF, Wu CY, Yeomans N, Vakil N, Goh KL, Lu Y, Loffroy R, Lau JYWW, Barkun A, Laine L, Jensen DM, Sung JJYY, Tsoi KKF, Lai LH, Wu JCY, Lau JYWW, Rotondano G, Hucl T, Dinis-ribeiro M, Marmo R, Racz I, Arezzo A, Sung JJYY, Chiu PCYWYCY, Chan FKLL, Lau JYWW, Goh KL, Ho LHYY, Jung HY, Sollano JD, Gotoda T, Reddy N, Singh R, Sugano K, Wu KC, Wu CY, Bjorkman DJ, Jensen DM, Kuipers EJ, Lanas A (2018). Asia-Pacific working group consensus on non-variceal upper gastrointestinal bleeding: an update 2018. Gut.

[30] Ang D, Teo EK, Tan A, Ibrahim S, Tan PS, Ang TL, Fock KM (2012). A comparison of surgery versus transcatheter angiographic embolization in the treatment of nonvariceal upper gastrointestinal bleeding uncontrolled by endoscopy. Eur J Gastroenterol Hepatol.

[31] Venclauskas L, Bratlie SO, Zachrisson K, Maleckas A, Pundzius J, Jönson C (2010). Is transcatheter arterial embolization a safer alternative than surgery when endoscopic therapy fails in bleeding duodenal ulcer?. Scand J Gastroenterol.

