# Supplementary material for: Reduced mortality for over-the-scope clips (OTSC) versus surgery for refractory peptic ulcer bleeding: a retrospective study
Source: Surg Endosc. 2022 Oct 17;37(3):1854–62. doi: 10.1007/s00464-022-09679-9 (PMC10017662; doi:10.1007/s00464-022-09679-9)
Supplement: Supplementary file 1 — Supplementary file1 (DOCX 20 kb) [file 464_2022_9679_MOESM1_ESM.docx]

**Supplementary Material**

**Suppl. Table 1**

| ICD-10-Codes - Ulcer | K25.0 ; K25.4 ; K26.0; K26.4 ; K27.0 ; K27.4 |
| --- | --- |
| OPS-Codes - OTSC | 5-449.d3, 5-449s ; 5-469.d3 , 5-469.s |
| OPS-Codes – Surgery | 5-433 ; 5-434 ; 5-435 ; 5-436 ; 5-437 ; 5-438 ; 5-439 ; 5-449.5 ; 5-451 ; 5-454.0 ; 5-454.4 ; 5-469.7 ; 5-469.8 |

**Supplementary Table 1:**

ICD-10 (International Statistical Classification of Diseases and Related Health Problems) and OPS codes that were used for the detection of patients in the hospital information system

**Suppl. Table 2**

| **Outcome stratisfied by shock** | **OTSC** | | **n = 29** | |  | |  | |  | **Surgery** | | **n = 25** | |  | |
| --- | --- | --- | --- | --- | --- | --- | --- | --- | --- | --- | --- | --- | --- | --- | --- |
| prim. hemostasis | | 27 | | 93,10 | |  | | [82,8-100,0] | | 24 | 96 | | [87,5-100,0] | | 0,999 |
| clin.success | | 22 | | 75,90 | |  | | [59,1-90,6] | | 20 | 80,00 | | [63,6-95,5] | | 0,755 |
| rebleeding during hospitalstay | | 5 | | 17,20 | |  | | [3,8-33,3] | | 5 | 20,00 | | [6,9-36,0] | | 0,999 |
| 7d rebleeding | | 5 | | 17,20 | |  | | [3,8-33,3] | | 4 | 16,00 | | [3,8-32,0] | | 0,999 |
| 30d rebleeding | | 5 | | 17,20 | |  | | [3,8-33,3] | | 5 | 20,00 | | [6,9-36,0] | | 0,999 |
| inhospital mortality | | 2 | | 6,90 | |  | | [0,0-17,6] | | 8 | 32,00 | | [14,8-52,2] | | **0,032** |
| 7d mortality | | 1 | | 3,40 | |  | | [0,0-11,5] | | 0 | 0,00 | |  | | 0,999 |
| 30d mortality | | 2 | | 6,90 | |  | | [0,0-17,6] | | 4 | 16,00 | | [3,7-32,0] | | 0,399 |
| rebleeding associated death | | 1 | | 3,40 | |  | | [0,0-11,5] | | 1 | 4,00 | | [0,0-12,5] | | 0,999 |

**Supplementary Table 2** Outcome parameters after stratification by presence of shock are shown. Statistical analysis was performed with Mann-Whitney U-Test (continuous variables), and χ^2^ tests or Fisher’s Exact tests (categorial variables). P values < 0.05 were considered being significant. OTSC = Over-The-Scope Clips, n = number, % = percentage, 95% CI = 95% confidence intervall
